# MicroRNA-16 sensitizes breast cancer cells to paclitaxel through suppression of IKBKB expression

**DOI:** 10.18632/oncotarget.8056

**Published:** 2016-03-14

**Authors:** Xueyuan Tang, Long Jin, Peiguo Cao, Ke Cao, Chenghui Huang, Yanwei Luo, Jian Ma, Shourong Shen, Ming Tan, Xiayu Li, Ming Zhou

**Affiliations:** ^1^ Department of Oncology, The Third Xiangya Hospital of Central South University, Changsha, Hunan, China; ^2^ Department of Gastroenterology, The Third Xiangya Hospital of Central South University, Changsha, Hunan, China; ^3^ The Key Laboratory of Carcinogenesis of The Chinese Ministry of Health and The Key Laboratory of Carcinogenesis and Cancer Invasion of The Chinese Ministry of Education, Cancer Research Institute, Central South University, Changsha, Hunan, China; ^4^ Hunan Key Laboratory of Nonresolving Inflammation and Cancer, Disease Genome Research Center, The Third Xiangya Hospital, Central South University, Changsha, Hunan, China; ^5^ Mitchell Cancer Institute, University of South Alabama, Mobile, Alabama, USA

**Keywords:** miR-16, IKBKB, Taxol, breast cancer, chemosensitivity

## Abstract

Paclitaxel (Taxol) is an effective chemotherapeutic agent for treating breast cancer patients. However, chemoresistance is a major obstacle in cancer treatment. Here, we showed that overexpression of miR-16 promoted Taxol-induced cytotoxicity and apoptosis in breast cancer cells. Furthermore, IκB kinase β (IKBKB) was identified as a direct target of miR-16. Up-regulation of IKBKB suppressed Taxol-induced apoptosis and led to an increased resistance to Taxol, and restoring IKBKB expression in miR-16-overexpressing breast cancer cells recovered Taxol resistance. Moreover, miR-16 was highly expressed in Taxol-sensitive breast cancer tissues compared with Taxol-resistant tissues, and there was an inverse correlation between miR-16 expression and IKBKB expression in breast cancer tissues. The expression levels of miR-16 were negatively associated with T stages, whereas the expression of IKBKB was positively correlated with T stages, lymph node metastasis and clinical stages. Taken together, our data demonstrates that miR-16 sensitizes breast cancer cells to Taxol through the suppression of IKBKB expression, and targeting miR-16/IKBKB axis will be a promising strategy for overcoming Taxol resistance in breast cancer.

## INTRODUCTION

Breast cancer is the most common malignancy in women and the leading cause of cancer-related death worldwide, with 232,340 new cases annually [1]. Paclitaxel (Taxol), an effective mitotic inhibitor, is commonly used in the treatment of breast cancer as well as other tumors, such as ovarian, prostate and non-small cell lung cancer [2,3]. The major mechanism underlying the anti-tumor activity of Taxol has been ascribed to its ability to interfere with microtubule dynamics through binding to the β-subunit of microtubule α–β tubulin heterodimer and inducing apoptosis by directly interacting with mitochondrial membrane proteins [4,5]. However, despite its widespread usage and infrequent side effects, the clinical efficacy of Taxol in the treatment of breast cancer is often compromised by the emergence of Taxol resistance, which typically appears following a couple cycles of Taxol based chemotherapy. The up-regulation of P-glycoprotein and related drug efflux pumps [6–9] and the altered expression of tubulin isotypes, particularly β III-tubulin [10,11], have been strongly implicated in Taxol resistance. However, the exact mechanisms responsible for the development of Taxol resistance remain elusive.

MicroRNAs (miRNAs) are endogenous, 18–25 nucleotide, non-coding, single-stranded RNA molecules, which play a critical role in a variety of biological processes, including proliferation, differentiation, migration, cell cycle and apoptosis [12,13]. In recent years, some miRNAs have been reported to be involved in drug resistance by acting as potential oncogenes or tumor suppressors [14–16]. MicroRNA-16 (miR-16) is located at 13q14, which also hosts the tumor suppressor miR-16-1/-15a cluster [17]. Further investigation indicated that miR-16 was frequently deleted or downregulated in solid tumors including breast cancer, which was identified as a tumor suppressor [18,19]. MiR-16 participates in cell-cycle regulation by targeting multiple cyclin proteins, and it induces apoptosis by targeting Bcl2 [20,21]. MiR-16 was found to be down-regulated by almost two-fold in Taxol-resistant breast cancer cells compared with their parental cells via miRNA microarray assays [14]. Therefore, we hypothesized that miR-16 may contribute to Taxol chemosensitivity in breast cancer.

In the present study, we demonstrated that ectopic expression of miR-16 promoted Taxol-induced cytotoxicity and apoptosis in breast cancer cells. Furthermore, IKBKB was identified to be a direct target of miR-16, restoring the expression of IKBKB counteracted miR-16-mediated Taxol sensitivity. Moreover, miR-16 was highly expressed in Taxol-sensitive breast cancer patients and negatively associated with T stages, whereas IKBKB was lowly expressed in Taxol-sensitive breast cancer and positively correlated with T, N and clinical stages. Taken together, these data indicate that miR-16 increases the chemosensitivity of breast cancer to Taxol through suppression of IKBKB expression, and combination of miR-16 targeted gene therapy and Taxol chemotherapy might represent a promising novel clinical strategy for human breast cancer.

## RESULTS

### Involvement of miR-16 in Taxol chemosensitivity in breast cancer cells

**Figure 1:**
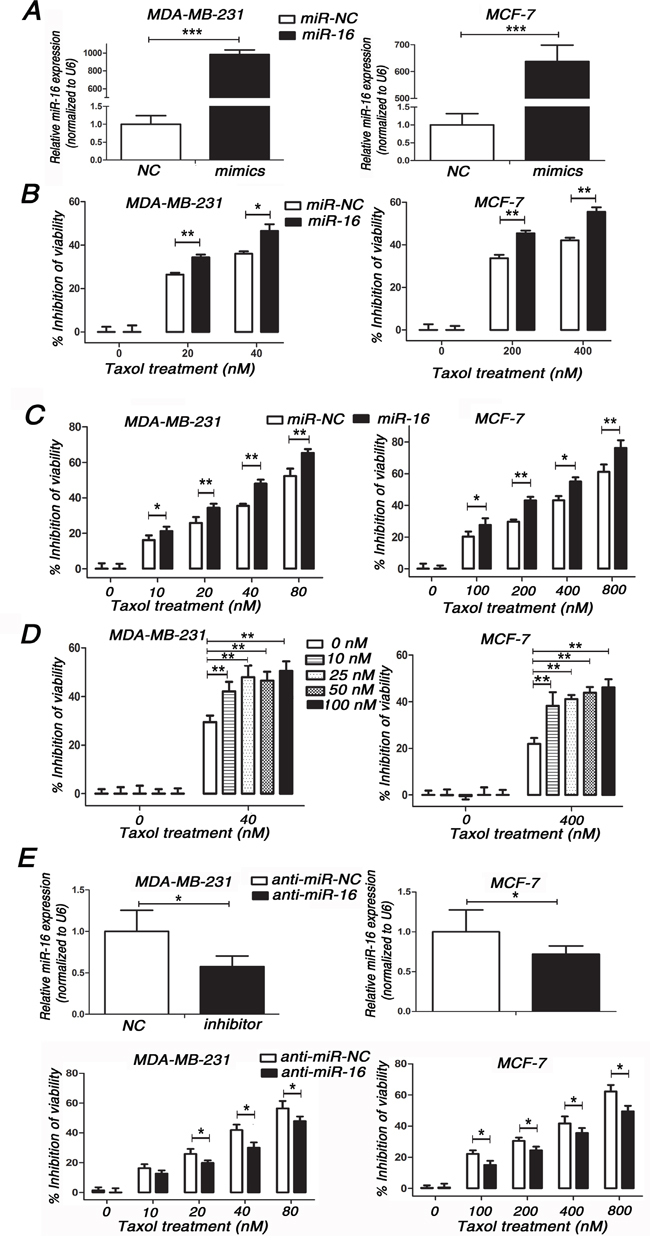
Involvement of miR-16 in Taxol chemosensitivity in breast cancer cells **A.** miR-16 levels were detected in MDA-MB-231 and MCF-7 cells transfected with 50 nM miR-16 mimics or 50 nM miR-NC by qRT-PCR. **B.** MDA-MB-231 and MCF-7 cells transfected with 50 nM miR-16 mimics or 50 nM miR-NC were seeded into 96-well plates and treated with 0, 20, 40 nM (MDA-MB-231) or 0, 200, 400 nM (MCF-7) Taxol for 48 h. The cell viabilities were detected by MTT assays. **C.** MDA-MB-231 and MCF-7 cells transfected with 50 nM miR-16 mimics or 50 nM miR-NC were seeded into 96-well plates and treated with 0, 10, 20, 40, 80 nM (MDA-MB-231) or 0, 100, 200, 400, 800 nM (MCF-7) Taxol for 48 h. The cell viabilities were detected by MTT assays. **D.** MDA-MB-231 and MCF-7 cells transfected with 0, 10, 25, 50, 100 nM miR-16 mimics or equal dose of miR-NC were seeded into 96-well plates and treated with 0, 40 nM (MDA-MB-231) or 0, 400 nM (MCF-7) Taxol for 48 h. The cell viabilities were detected by MTT assays. **E.** MDA-MB-231 and MCF-7 cells transfected with 100 nM miR-16 inhibitor or 100 nM anti-miR-NC (top) were seeded into 96-well plates and treated with 0, 10, 20, 40, 80 nM (MDA-MB-231) or 0, 100, 200, 400, 800 nM (MCF-7) Taxol for 48 h. The cell viabilities were detected using MTT assays. Data are presented as the percentage of viability inhibition measured in untreated cells. Columns, means of three independent experiments; bars, SE. *, p<0.05, **, p<0.01, ***, p<0.001.

To investigate whether the overexpression of miR-16 could sensitize breast cancer cells to Taxol, miR-16 mimics (miR-16) were transfected into two breast cancer cell lines, MDA-MB-231 (Figure 1A, left) and MCF-7 (Figure 1A, right), followed by treatment with Taxol for 48 h. We found that overexpression of miR-16 markedly increased Taxol-induced cell cytotoxicity in both MDA-MB-231 (Figure 1B, left) and MCF-7 cells (Figure 1B, right) compared with the negative control (miR-NC). To confirm and extend our findings, we treated these miR-16-transfected cells with a dose range of Taxol and obtained the similar results in both cell lines (Figure 1C, Figure S1A), and the IC50 values of miR-16-transfected cells were much lower than miR-NC-transfected and mock cells (Figure S1B). Moreover, we found there was a trend that overexpression of miR-16 dramatically enhanced the cytotoxicity of Taxol and inhibited Taxol resistance in a miR-16 dose-dependent manner in both MDA-MB-231 (Figure 1D, left) and MCF-7 cells (Figure 1D, right). These results demonstrate a positive correlation between miR-16 expression and Taxol response in breast cancer cells.

Since overexpression of miR-16 increased Taxol sensitivity, we wondered whether down-regulation of miR-16 would confer breast cancer cells resistant to Taxol. Thus, the miR-16 inhibitor (anti-miR-16) was transiently transfected in MDA-MB-231 (Figure 1E, top left) and MCF-7 cells (Figure 1E, top right) followed by treatment with increasing concentrations of Taxol for 48 h. As expected, the knockdown of miR-16 impaired Taxol-induced cytotoxicity in these two cell lines (Figure 1E, bottom). These results demonstrate that miR-16 plays an important role in Taxol-induced chemosensitivity in breast cancer cells.

### Involvement of miR-16 in Taxol-induced apoptosis in breast cancer cells

**Figure 2:**
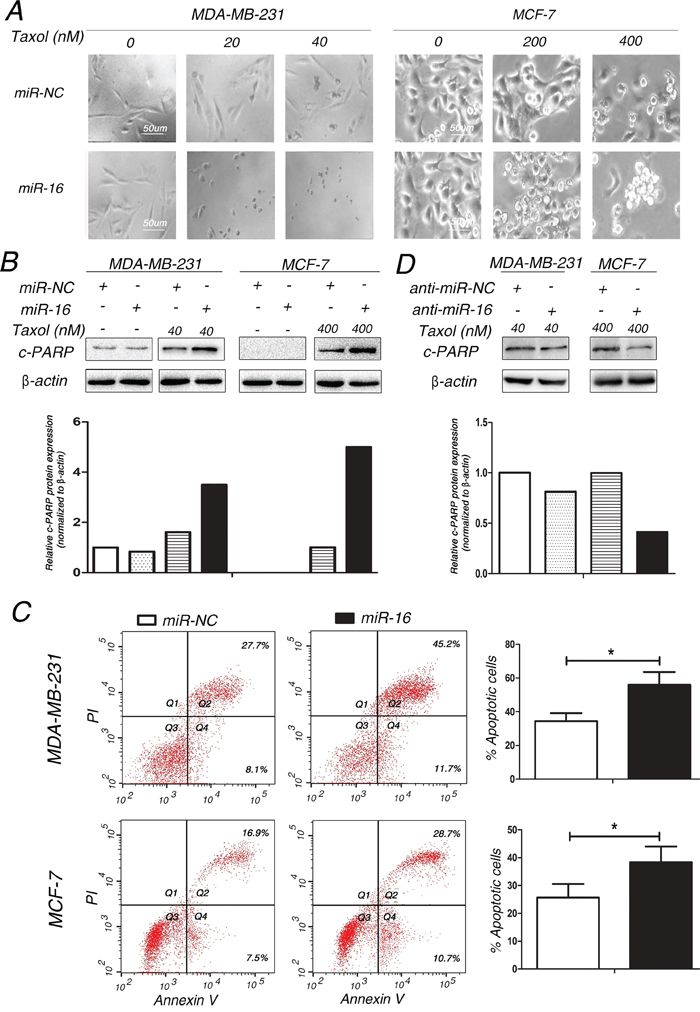
Involvement of miR-16 in Taxol-induced apoptosis in breast cancer cells **A.** MDA-MB-231 and MCF-7 cells transfected with 50 nM miR-16 mimics or 50 nM miR-NC were treated with 0, 20, 40 nM (MDA-MB-231) or 0, 200, 400 nM (MCF-7) Taxol for 48 h. The cellular morphologies were visualized using a phase-contrast microscope. **B-C.** MDA-MB-231 and MCF-7 cells were transfected with 50 nM miR-NC or miR-16 mimics and then treated with 40 and 400 nM Taxol for 48 h, respectively. Cell lysates were extracted for western blotting using an antibody against c-PARP (B), or cells were collected for annexin V staining and flow cytometry assays (C). The gray density was quantified using the ImageJ software and normalized to β-actin. The percentage of apoptotic cells is represented in a bar diagram from three independent experiments. **D.** MDA-MB-231 and MCF-7 cells transfected with 100 nM anti-miR-NC or miR-16 inhibitor were treated with 40 and 400 nM Taxol for 48 h, respectively. Cell lysates were extracted for western blotting using an antibody against c-PARP. β-actin was used as an internal control. Columns, means of three independent experiments; bars, S.E. *, p<0.05, **, p<0.01.

Taxol is known to exert its anti-tumor effect through induction of apoptosis [22]. Next, we examined whether overexpression of miR-16 was capable of enhancing Taxol-induced apoptosis. Our results showed that more cellular debris and non-viable cells were observed with an inverted microscope in miR-16-transfected MDA-MB-231 (Figure 2A, left) and MCF-7 (Figure 2A, right) cells compared with the control groups after treatment of Taxol. Poly (ADP-ribose) polymerase (PARP) is an important marker of apoptosis that is cleaved by caspases during cellular apoptosis [23,24]. Therefore, we detected the protein levels of cleaved PARP (c-PARP) by western blot assays in miR-16-transfected or miR-NC-transfected MDA-MB-231 and MCF-7 cells after Taxol treatment. Compared with the negative control, the protein levels of c-PARP were dramatically increased in miR-16-transfected cells (Figure 2B). In line with the former results, apoptosis assays with annexin V staining revealed more apoptotic cells in miR-16-transfected cells after treatment of Taxol (Figure 2C). Conversely, knockdown of endogenous miR-16 impaired Taxol-induced apoptosis in these two cell lines by comparing the protein levels of c-PARP (Figure 2D). These results indicate that miR-16 enhances Taxol sensitivity in breast cancer cells by promoting Taxol-induced apoptosis.

### IKBKB is a direct target of miR-16 in breast cancer cells

**Figure 3:**
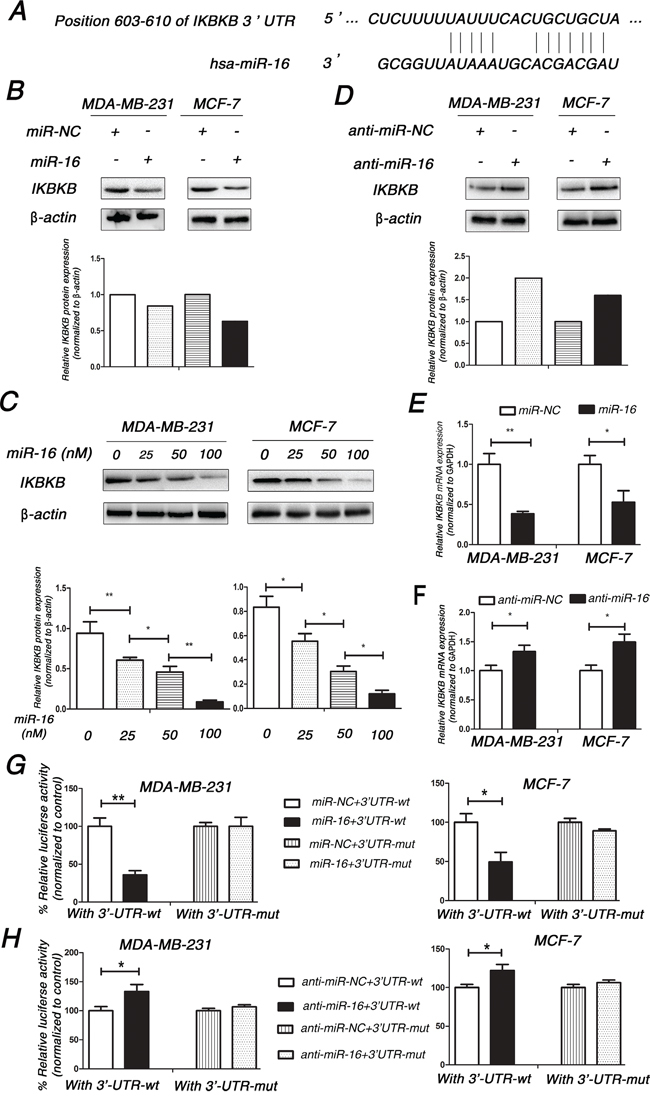
IKBKB is a direct target of miR-16 in breast cancer cells **A.** Schematic description of interaction between IKBKB and miR-16. **B.** MDA-MB-231 and MCF-7 were transfected with 50 nM miR-NC or miR-16 mimics. Cell lysates were prepared for western blotting with an antibody against IKBKB 48 h after transfection. β-actin was used as a loading control. The gray density was quantified using the ImageJ software and normalized to β-actin. **C.** 0, 25, 50, 100 nM miR-16 mimics were transfected into MDA-MB-231 and MCF-7 cells, and then the expression of IKBKB was detected by western blotting assay as (B) performed. **D.** MDA-MB-231 and MCF7 were transfected with 100 nM anti-miR-NC or miR-16 inhibitor, and then similar experiments as in (B) were performed. **E.** MDA-MB-231 and MCF-7 were transfected with 50 nM miR-NC or miR-16 mimics. Total cellular RNA was collected and IKBKB mRNA levels were measured by qRT-PCR 24 h after transfection. The relative mRNA levels of IKBKB were shown in the bar diagram from three independent experiments and normalized to GAPDH. **F.** MDA-MB-231 and MCF-7 were transfected with 100 nM anti-miR-NC or miR-16 inhibitor, and similar experiments as in (E) were performed and analyzed. **G.** MDA-MB-231 and MCF-7 were co-transfected with pMIR-IKBKB-3′UTR-wt or pMIR-IKBKB-3′UTR-mut and 50 nM miR-16 mimics using Lipofectamine 3000 reagent. Luciferase activity was measured 48 h after transfection. The pRL-TK vector was used as an internal control. The results were expressed as relative luciferase activity (firefly luc/renilla luc). **H.** MDA-MB-231 and MCF-7 cells were co-transfected with pMIR-IKBKB-3′UTR-wt or pMIR-IKBKB-3′UTR-mut and 100 nM miR-16 inhibitor using Lipofectamine 3000 reagent, and similar experiments as in (G) were performed and analyzed. Columns, means of three independent experiments; bars, S.E. *, p<0.05, **, p<0.01.

To elucidate the mechanisms underlying promoting the response of human breast cancer cells to Taxol by miR-16, we used three bioinformatic programs (TargetScan, miRDB and miRanda) to predict the potential target genes of miR-16. All of the three public miRNA databases predicted that IKBKB might be a potential target for miR-16, and the 3′-UTR of IKBKB contains a highly conserved binding site for miR-16 from position 603 to 610 (Figure 3A). We further demonstrated that the overexpression of miR-16 significantly down-regulated the protein level of IKBKB in a dose-dependent manner in MDA-MB-231 and MCF7 cells (Figure 3B and 3C). In contrast, knockdown of miR-16 up-regulated the expression of IKBKB in both cell lines (Figure 3D). Next, qPCR was performed to investigate the effect of miR-16 on IKBKB mRNA expression in MDA-MB-231 and MCF-7 cells. As expected, overexpression of miR-16 significantly decreased the expression of IKBKB mRNA (Figure 3E), whereas knockdown of miR-16 increased the mRNA levels of IKBKB compared with control cells in both MDA-MB-231 and MCF7 cell lines (Figure 3F). The above results support that miR-16 could negatively regulate the expression of IKBKB at both mRNA and protein levels.

As miR-16 could negatively regulate the expression of IKBKB, we wanted to know whether miR-16 could directly target IKBKB. Therefore, dual-luciferase reporter analysis was performed to detect whether miR-16 directly bound to the 3′-UTR of IKBKB mRNA. As shown in Figure 3G, overexpression of miR-16 decreased the luciferase activity of the reporter gene with wild-type 3′-UTR (3′-UTR-wt) of IKBKB, while no inhibitory effect was detected with the mutated 3′-UTR (3′-UTR-mut) of IKBKB. On the contrary, knockdown of miR-16 increased the luciferase activity of the reporter gene with wild-type 3′-UTR (3′-UTR-wt) of IKBKB, while no inhibitory effect was detected with the mutated 3′-UTR (3′-UTR-mut) of IKBKB (Figure 3H). These data support that IKBKB is a direct target of miR-16 in breast cancer cells.

### IKBKB plays a critical role in Taxol-induced apoptosis

**Figure 4:**
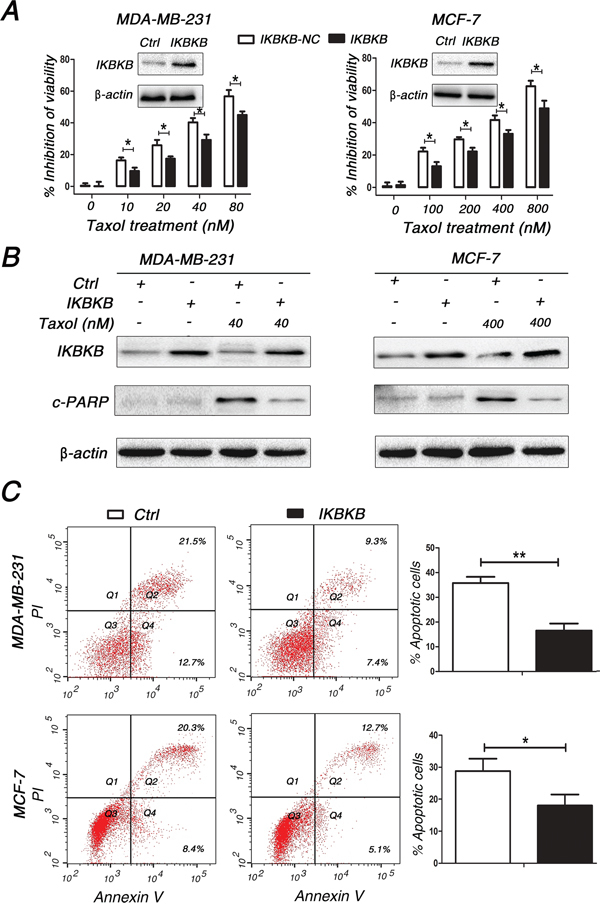
IKBKB plays a critical role in Taxol-induced apoptosis **A.** MDA-MB-231 and MCF-7 cells transfected with blank-plasmid or IKBKB (inset) overexpression plasmid were seeded into 96-well plates and treated with 0, 10, 20, 40, 80 nM (MDA-MB-231) or 0, 100, 200, 400, 800 nM (MCF-7) Taxol for 48 h. The inhibition of cell viabilities was detected using MTT assays. Columns, means of three independent experiments; bars, S.E. *p<0.05. **B-C.** MDA-MB-231 and MCF-7 cells were transfected with blank-plasmid or IKBKB overexpression plasmid and then treated with 40 and 400 nM Taxol for 48 h, respectively. Cell lysates were extracted for western blotting using antibodies against c-PARP and IKBKB (B), or cells were collected for annexin V staining and flow cytometry assays (C). The percentage of apoptotic cells is represented in a bar diagram from three independent experiments (C, right). β-actin was used as a loading control. Columns, means of three independent experiments; bars, S.E. *, p<0.05, **, p<0.01.

IKBKB, a component of NF-κB signaling, has been proven to participate in apoptosis [25]. However, its role in Taxol-induced cell cytotoxicity and apoptosis is not well understood. Thus, we transfected an IKBKB overexpression plasmid into MDA-MB-231 (Figure 4A, left inset) and MCF-7 (Figure 4A, right inset) cells and subsequently treated these cells with a dose range of Taxol. Our results demonstrated that the overexpression of IKBKB efficiently inhibited Taxol-induced cell cytotoxicity (Figure 4A). Furthermore, cleaved PARP was dramatically reduced in IKBKB-transfected MDA-MB-231 and MCF-7 cells after treatment with Taxol for 48 h (Figure 4B), suggesting that overexpression of IKBKB suppressed Taxol-induced cell apoptosis. These data were further validated by annexin V staining (Figure 4C). These results indicates that overexpression of IKBKB could suppress Taxol-induced cell cytotoxicity and apoptosis.

### Restoring IKBKB expression counteracts miR-16-mediated Taxol sensitivity

**Figure 5:**
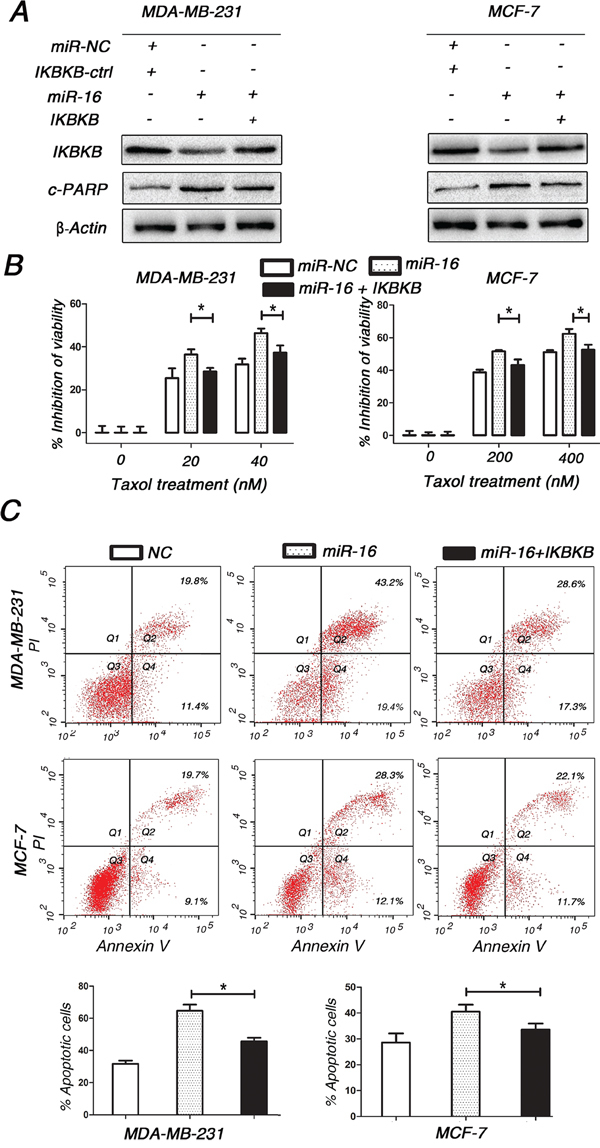
Restoring the expression of IKBKB recovers Taxol resistance and counteracts miR-16-mediated Taxol sensitivity **A.** MDA-MB-231 and MCF-7 cells were transfected with miR-16 mimics alone or miR-16 mimics plus IKBKB overexpression plasmid and then treated with 40 and 400 nM Taxol for 48 h, respectively. Cell lysates were extracted for western blotting using antibodies against c-PARP and IKBKB. β-actin was used as a loading control. **B.** MDA-MB-231 and MCF-7 cells transfected with miR-16 mimics alone or miR-16 mimics plus IKBKB overexpression plasmid were seeded into 96-well plates and treated with 0, 20, 40 nM (MDA-MB-231) or 0, 200, 400 nM (MCF-7) Taxol for 48 h. The cell viabilities were then detected using MTT assays. Columns, means of three independent experiments; bars, S.E. *, p<0.05. **C.** MDA-MB-231 and MCF-7 cells were transfected with miR-16 mimics alone or miR-16 mimics plus IKBKB overexpression plasmid and then treated with 40 and 400 nM Taxol for 48 h, respectively. Cells were collected for annexin V staining and flow cytometry assays. Percentages of apoptotic cells are represented in bar diagram from three independent experiments. Columns, means of three independent experiments; bars, S.E. *, p<0.05.

Since IKBKB plays a critical role in Taxol-induced cell cytotoxicity and apoptosis, we sought to determine whether miR-16 promoted the response of breast cancer cells to Taxol by suppressing IKBKB expression. Compared with cells transfected with miR-16 mimics alone, MDA-MB-231 and MCF-7 cells transfected with miR-16 mimics plus IKBKB overexpression vector partially restored Taxol-induced cytotoxicity (Figure 5B) and apoptosis (Figure 5A and 5C). Taken together, these results clearly demonstrate that miR-16 could sensitize breast cancer cells to Taxol at least partially through the direct suppression of IKBKB expression.

### Higher expression of miR-16 was detected in Taxol-sensitive breast cancer tissues compared with Taxol-resistant tissues

**Figure 6:**
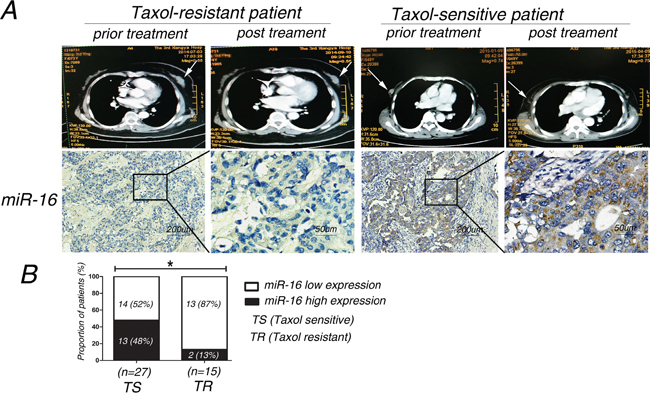
The expression level of miR-16 in Taxol-sensitive breast cancer tissues is higher than that in Taxol-resistant tissues **A.** miR-16 expression was measured by in situ hybridization in paraffin-embedded Taxol-treated breast cancer tissues. Representative cases of Taxol-sensitive (n=27) and Taxol-resistant (n=15) patients with high or low miR-16 staining are shown. **B.** Proportion of Taxol-treated breast cancer patients with low or high miR-16 expression. *, p<0.05.

Since miR-16 was associated with responses of breast cancer cells to Taxol, we wondered whether there was a difference in miR-16 expression levels between Taxol-sensitive and Taxol-resistant breast cancer tissues. Therefore, in situ hybridization (ISH) was performed to investigate the expression levels of miR-16 in paraffin-embedded breast cancer samples. Computed tomography (CT) changes before and after Taxol treatment were used to differentiate Taxol-sensitive and resistant patients (Figure 6A). Our results reveal that the expression of miR-16 was higher in 27 Taxol-sensitive breast cancer samples than in the 15 Taxol-resistant samples (48%, 13/27 vs. 13%, 2/15; p < 0.05; Figure 6A and B).

### The association of miR-16 and IKBKB expression with the clinicopathological characteristics of breast cancer patients

**Figure 7:**
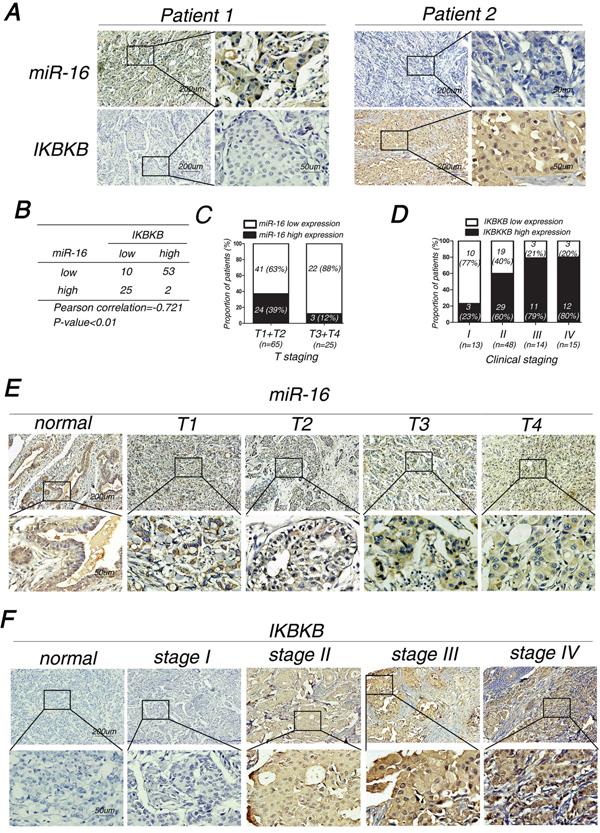
MiR-16 expression is inversely correlated with IKBKB expression and their expression levels are associated with clinicopathological characteristics of breast cancer patients **A.** Representative images of inverse correlation between miR-16 and IKBKB expression are shown. **B.** Spearman rank test of 90 breast cancer patients was used for depicting the correlation between miR-16 and IKBKB. **C-D.** Proportion of breast cancer patients with miR-16 expression and T stages (C) or IKBKB expression and clinical stages (D). **E-F.** Representative images of breast cancer patients with miR-16 expression and T stages (E) or IKBKB expression and clinical stages (F).

As miR-16 could directly target IKBKB in breast cancer cells, we wondered whether miR-16 expression was negatively associated with IKBKB expression in breast cancer tissues. Therefore, we detected the expression of miR-16 and IKBKB in paraffin-embedded breast cancer samples by in situ hybridization (ISH) and immunohistochemical (IHC) analysis, respectively. We found that miR-16 and IKBKB expression were predominantly positive in the cytoplasm, while they were rarely expressed in the nuclei. Moreover, a significant inverse association was observed between miR-16 and IKBKB expression in breast cancer specimens (Figure 7A and B). We then analyzed miR-16 and IKBKB expression and their possible associations with clinicopathological parameters, such as age, pathological classification, pathological grade, tumor size (T staging), lymph node metastasis (N staging), distant tumor metastasis (M staging) and clinical staging (Table 1). The data indicated that miR-16 expression was negatively associated with T staging (Table 1; Figure 7C and 7E), but it presented a non-significant association with age, pathological classification, pathological grade, N, M and clinical staging. Meanwhile, IKBKB expression was positively correlated with T, N and clinical staging (Table 1; Figure 7D and 7F), but it was non-significantly correlated with age, pathological classification, pathological grade, and M staging.

**Table 1:** Associations between miR-16 and IKBKB expression levels and the clinicopathological characteristics of breast cancer patients

Variables	Cases (n)	miR-16 Expression (n)		P value	IKBKB Expression (n)		P value
		Low	High		Low	High	
Age (years)				0.129			0.060
<50	56	36	20		26	30	
≥50	34	27	7		9	25	
Pathological classification				0.275			0.351
Invasive ductal carcinoma	59	44	15		20	39	
Invasive lobular carcinoma	16	11	5		7	9	
Medullary carcinoma	15	8	7		8	7	
Pathological grade *				0.953			0.663
I	8	6	2		3	5	
II	37	28	9		11	26	
III	14	10	4		6	8	
T staging				0.021			<0.001
T1+T2	65	41	24		34	31	
T3+T4	25	22	3		1	24	
Lymph node metastasis				0.059			<0.001
No	43	26	17		27	16	
Yes	47	37	10		8	39	
Distant metastasis				0.537			0.100
No	75	51	24		32	43	
Yes	15	12	3		3	12	
Clinical staging				0.711			0.008
I	13	8	5		10	3	
II	48	34	14		19	29	
III	14	9	5		3	11	
IV	15	12	3		3	12	

* The correlation between miR-16/IKBKB expression and pathological grade *were only* analyzed in *59 cases of Invasive ductal carcinoma patients*.

## DISCUSSION

Taxol based combination chemotherapy is widely used to treat and extend survival in patients diagnosed with breast cancer [2]. MiR-16 was reported to be involved in chemoresistance of anti-cancer drugs in multiple of caner types by targeting different genes [26,27]. Cittelly et al showed that miR-16 could directly down-regulate Bcl-2, subsequently sensitizing breast cancer to the endocrine drug tamoxifen [26]. Huang N et al found that miR-16 could enhance the chemosensitivity of camptothecin by directly targeting Rictor through autophagy signaling in HeLa cells [27]. In our study, miR-16 was found to enhance Taxol-induced cytotoxicity and chemosensitivity in breast cancer cells. As Taxol is known to exert its anti-tumor effect through induction of apoptosis [22], we further demonstrated that although miR-16 has no significant effect on apoptosis in breast cancer cells (Figure S2), miR-16 could increase Taxol-induced apoptosis, and this pro-apoptotic effect was a key mechanism of miR-16-mediated Taxol sensitivity in breast cancer cells. Previous studies have also demonstrated that miR-16 could induce apoptosis in chronic lymphocytic leukemia (CLL) [28].

IκB kinase β (IKBKB) was identified as a component of NF-κB signaling and is highly active in various malignancies including acute myeloid leukemia, melanoma, breast and pancreatic cancer [29–32]. The breast cancer MDA-MB-453 cell line with ectopic expression of IKBKB has a higher proliferation rate than its IKBKB-negative counterpart [33]. Song et al validated that miR-218 inhibited the invasive ability of glioma cells through the direct downregulation of IKBKB [34], suggesting that IKBKB could effectively regulate the behaviors of cancer cells. In accordance with previous data, we identified IKBKB as a direct target of miR-16, overexpression of IKBKB could decrease Taxol-induced cytotoxicity and apoptosis in breast cancer cells. To our knowledge, this is the first report to demonstrate a direct link between IKBKB expression and Taxol sensitivity in cancer cells. Furthermore, our results validated an inverse correlation between miR-16 expression and IKBKB expression in breast cancer tissues. Importantly, we found that miR-16 contributed to Taxol chemosensitivity through the suppression of IKBKB expression, which partially explained miR-16-mediated Taxol cytotoxicity, indicating that the miR-16-IKBKB axis was involved in Taxol sensitivity in breast cancer cells. Although restoring the expression of IKBKB could only partially abrogate Taxol-induced cell cytotoxicity and apoptosis, this might be explained by the diversity of miR-16 target genes, which collectively conferred miR-16-mediated Taxol sensitivity. These novel findings provide a unique insight into the molecular mechanism of miR-16-mediated Taxol sensitivity.

NF-κB pathway is critical for the normal development of mammary gland by promoting proliferation, motility and invasion [35], abnormal activation of NF-κB pathway can be observed in breast cancer [36–39]. In addition, NF-κB activation has been also implicated in cancer chemotherapy resistance mechanisms [40]. And inhibition of NF-κB pathway by either the IKK inhibitor PA or the proteasome inhibitor PS-341 is able to restore the anti-estrogenic effects of tamoxifen in breast cancer cells [41,42]. In the classical pathway, IKBKB activates NF-κB (p65) through phosphorylation of inhibitors of NF-κB (IκBs), thus leading to p65 nucleus translocation [43] and resulting in subsequent transcription of downstream genes involved in tumorigenesis and anti-apoptosis [44] including anti-apoptotic (Bcl-2, Bcl-xL, survivin), proliferative (cyclin D1), proinflammatory (COX-2), invasive (MMP-9) and angiogenic (VEGF) genes. In our study, the protein level of p-p65 and NF-κB activation were found to be decreased after overexpression of miR-16 in breast cancer cells with Taxol treatment (Figure S3A and S3B). We propose that miR-16 may promote apoptosis via an IKBKB-p65-Bcl-2 axis, resulting in chemosensitivity to Taxol, which still need further investigations and large patient samples to illustrate it.

To date, miR-16 has been identified to participate in tumorigenesis, development and progression, and it has been shown to be a tumor suppressor in different tumors [45–47]. Especially, miR-16 was validated to decrease cellular growth and proliferation and induce apoptosis in MCF-7 cells [47], implying that miR-16 might be involved in breast cancer development and progression. To confirm this hypothesis, we detected expression levels of miR-16 and IKBKB in breast cancer samples and found that miR-16 was negatively associated with T stages, while IKBKB positively correlated with T, N and clinical stages, which supported that miR-16 and IKBKB might participate in tumorigenesis of breast cancer.

Taken together, we have shown that miR-16 is positively involved in Taxol-induced cell cytotoxicity and apoptosis in breast cancer cells. IKBKB was identified as a direct target of miR-16, restoring the expression of IKBKB in miR-16-ovexpressing breast cells could recover Taxol resistance. Furthermore, an inverse correlation between miR-16 and IKBKB expression was found in breast cancer tissues, miR-16 was negatively associated with T stages, whereas IKBKB was positively correlated with T, N and clinical stages. Therefore, miR-16 sensitizes breast cancer cells to Taxol through the suppression of IKBKB expression, and it may potentially serve as a therapeutic target for overcoming Taxol resistance in human breast cancer.

## MATERIALS AND METHODS

### Cells and human tissues

Human breast cancer cell lines MDA-MB-231 and MCF-7 were maintained in our laboratory. MDA-MB-231 cells were cultured in RPMI 1640 medium, and MCF-7 cells were cultured in DMEM medium in a humidified incubator with 5% CO_2_ at 37°C. Cell media was supplemented with 10% fetal bovine serum (FBS, Invitrogen, Shanghai, China) and penicillin/streptomycin.

All tissue samples were collected from diagnosed breast cancer patients who were confirmed by histopathological examination at the Third Xiangya Hospital (Changsha, China) from 1st Jan. 2004 to 30th Jun. 2015. Among the selected cases, 42 of the patients received Taxol-combined neoadjuvant chemotherapy and their sensitivity or resistance to Taxol were differentiated by computed tomography (CT) analysis before and after Taxol treatment, of which, 25 cases presented Taxol-sensitive and 17 cases resistant.

This study was approved by the Research Ethics Board of the Third Xiangya Hospital and signed informed consent was obtained from each participant before they were enrolled in the study.

### Overexpression and knockdown of miR-16

MDA-MB-231 and MCF-7 cells were seeded in 6-well plates, and on the following day, when the cells were approximately 70% confluent, they were transfected with 50 nM miR-16 mimics (miR-16), 100 nM miR-16 inhibitor (anti-miR-16), or equal amounts of scrambled negative control RNA (Ribobio, Guangzhou, China) using Lipofectamine 3000 (Invitrogen, Guangzhou, China). The cells were then harvested 24 h after transfection for quantitative RT-PCR and 48 h after transfection for western blot assays.

### Plasmid construction and overexpression of IKBKB

A mammalian expression plasmid encoding the human IKBKB open reading frame (pGV219-IKBKB) was purchased from Genechem (Shanghai, China). An empty plasmid was served as a negative control. IKBKB expression plasmid and negative plasmid (2 μg) were transfected into MDA-MB-231 and MCF-7 cells using Lipofectamine 3000 according to the manufacturer's instructions. Total RNA was isolated 24 h post-transfection for quantitative RT-PCR, and protein was extracted 48 h after transfection for the western blot assay.

### RNA isolation and quantitative RT-PCR

Total RNA was extracted from cultured cells using TRIzol Reagent (Invitrogen, Shanghai, China) according to the manufacturer's instructions. Assays to quantify miRNAs were performed using the miRNA qRT-PCR Kit (Ribobio, Guangzhou, China). Briefly, 1 μg of total RNA was added with Poly(A)-Tailing and reverse-transcribed to cDNA using Reverse Transcriptase and Uni-Reverse Primer (Ribobio). The reaction conditions were as follows: 37¼C for 60 min, 42¼C for 60 min, and 72¼C for 10 min. Real-time PCR was performed using specific primers for miR-16 and U6 under the following conditions: 95¼C for 10 min, followed by 40 cycles of 95¼C for 10 s, 60¼C for 20 s, and 70¼C for 1 s. The sequences of the primers were as followings: miR-16, 5′ primer (5-TAGCAGCACGTAAATATTGGCG-3) and 3′ primer (provided by Ribobio); U6, 5′ primer (5-ATTGGAACGATACAGAGAAGATT-3) and 3′ primer (5-GGAACGCTTCACGAATTTG-3). All of the reactions were run in triplicate and the relative levels of miR-16 were normalized to U6.

To detect IKBKB mRNA levels, 2 μg of total RNA was reverse-transcribed to cDNA using oligo(dT) and the Thermoscript kit (Thermo Scientific, Shanghai, China) under the following conditions: 42¼C for 60 min and 70¼C for 5 min. Next, real-time PCR was performed using the RT product, SYBER Green Dye (TaKaRa, Dalian, China) and specific primers for IKBKB and GAPDH. The sequences of the primers were as followings: IKBKB, 5′ primer (5-TGAGAAGACTGTTGTCCGGC-3) and 3′ primer (5-GCAGGGTGCAGAGGTTATGT-3); GAPDH, 5′ primer (5-CGAGATCCCTCCAAAATCAA-3) and 3′ primer (5-TTCACACCCATGACGAACAT-3). The reactions were incubated at 95¼C for 3 min, followed by 40 cycles of 95¼C for 10 s, 60¼C for 30 s, and 72°C for 30 s. All of the reactions were run in triplicate and the relative amount of IKBKB mRNA was normalized to GAPDH.

### Protein extraction and western blot assay

All cells were rinsed with PBS (pH 7.4) and lysed in RIPA Lysis buffer (Beyotime, China) supplemented with a protease and phosphatase inhibitor Cocktail (Thermo Scientific 78440) on ice for 30 min. Cell lysates were cleared by centrifugation at 14,000 g at 4¼C for 20 min. Supernatants were collected, and protein concentrations were calculated using a Pierce BCA protein assay kit (Thermo Scientific). Proteins samples (50 μg) were separated by 10% sodium dodecylsulfate-polyacrylamide gel electrophoresis (SDS-PAGE) and then transferred onto a PVDF membrane. Membranes were incubated overnight at 4°C with primary anti-IKBKB (dilution 1:200, #20979-1-AP, Proteintech, Wuhan, China) and anti-cleaved PARP (dilution 1:1000, #5625, Cell Signaling, Shanghai, China) antibody. The next day, Membranes were washed with PBS and then incubated with a horseradish peroxidase-conjugated secondary antibody for 1 h at room temperature. The signal was visualized using an ECL detection reagent and quantified by densitometry using the Image J software (http://rsb.info.nih.gov/ij). Beta-actin was used as a loading control and detected using mouse anti-β-Actin antibody (dilution 1:2000, #AM10128, Abgent, Suzhou, China).

### Cell proliferation assay

MDA-MB-231 (8×10^3^/well) and MCF-7 (5×10^3^/ well) cells were cultivated in 96-well plates and treated with Taxol for 48 h. MTT solution (0.5 mg/ml) was added to each well and incubation was continued for 4 h at 37°C. DMSO (100 μl. Sigma) was then added to solute the dye and the absorbance was recorded at 490 nm. All tests were performed in triplicate. The inhibitory rate of Taxol-treated cells was normalized to that of Taxol-untreated cells and calculated by the following equation: inhibitory rate of cells = ((OD of untreated wells−OD of treated wells) / OD of untreated wells)× 100%.

### Apoptosis assay

MDA-MB-231 and MCF-7 cells were transfected with miR-16 mimics, miR-16 inhibitor, IKBKB overexpression plasmid, or miR-16 mimics plus IKBKB overexpression plasmid and then treated with indicated concentrations of Taxol for 48 h to induce apoptosis. The miR-control, anti-miR-control and control plasmid were served as negative controls. Both of the attached and floating cells were harvested and flow cytometry analysis was performed using an Annexin V-FITC/PI staining kit (BD Biosciences, CA, USA) according to the manufacturer's instructions. All experiments were performed in triplicate.

### Luciferase reporter assay

For the luciferase reporter assay of miRNA binding site, the wild-type or mutant IKBKB 3′-UTRs (from UGCUGCU to ACGACGA) were cloned into the pMIR-reporter plasmid immediately downstream of the firefly luciferase gene. All of the luciferase constructs were verified by sequencing. MDA-MB-231 and MCF-7 cells were transiently co-transfected with pMIR-IKBKB-3′UTR-wt or pMIR-IKBKB-3′UTR-mut, renilla luciferase plasmid (pRL-TK vector), miR-16 mimics or miR-16 inhibitor using Lipofectamine 3000 transfection reagent according to the manufacturer's protocol. After 48 h of transfection, cells were harvested and luciferase activities were determined using the Promega dual luciferase reporter assay kit. pRL-TK vector was used as the internal control. The relative luciferase activities were calculated by the ratio of firefly luc/ renilla luc activity and normalized to that of the control cells.

As for the luciferase reporter assay of NF-кB regulation, MDA-MB-231 and MCF-7 were co-transfected with 50 nM miR-Neg or miR-16 mimics, pNF-кB-Luc construct (stored in our lab) and pRL-TK plasmid (Promega, Madison, WI, USA) using Lipofectamine 3000 reagent. Luciferase activity was measured after treatment with 40 nM (MDA-MB-231) or 400 nM (MCF-7) Taxol for 24 h, respectively. The pRL-TK was used as an internal control. The results were expressed as relative luciferase activity (firefly luc/renilla luc).

### In situ hybridization (ISH) analysis

In situ hybridization was performed to detect miR-16 expression in tissue specimens. The probes were synthesized and labeled with DIG-dUTP at the 5′ end (Sangon Biotech, Shanghai, China). Hybridization, washing, and scanning were performed according to the manufacturer's instructions. Two independent pathologists who were blinded to the clinicopathological information scored the samples. The staining intensity was scored as 0 (negative), 1 (+), 2 (++), and 3 (+++). The extent of staining was scored as 0~1.0 (0%~100%). The final staining score (0–3) was calculated as the multiplication of the intensity score and extent score. The final score ≥ 1 was defined as high expression, otherwise was defined as low expression [48].

### Immunohistochemical (IHC) staining

IHC staining was performed using an UltraSensitive SP Kit (Maixin Biotechnology Company, Fuzhou, China). The staining was developed using DAB as the chromogen. The slides were counterstained with Mayer's hematoxylin and mounted for evaluation under a microscope. Two independent pathologists who were blinded to the clinicopathological information scored the samples. The score was judged by the multiplication of the intensity score and extent score similar to that used in the in situ hybridization analysis described above.

### Statistical analysis

Statistical analysis was performed by using SPSS software, version 19.0 (SPSS, Chicago, IL, USA) and GraphPad Prism 5. Student's *t*-test was used to evaluate significant differences between any two groups of data, and one way ANOVA test was used to evaluate significant differences for multiple comparisons. Kruskal-Wallis H test was used to evaluate significant differences for ranked data. Correlation analysis was performed using the Spearman method. Data were shown as the means ± standard error (S.E.) The value of p < 0.05 was considered significant.

## SUPPLEMENTARY FIGURES


