# Supplementary material for: MicroRNA-16 sensitizes breast cancer cells to paclitaxel through suppression of IKBKB expression
Source: Oncotarget. 2016 Mar 14;7(17):23668–83. doi: 10.18632/oncotarget.8056 (PMC5029655; doi:10.18632/oncotarget.8056)
Supplement: Supplementary file 1 [file oncotarget-07-23668-s001.pdf]

MicroRNA-16 sensitizes breast cancer cells to paclitaxel through suppression of IKBKB expression

SUPPLEMENTARY FIGURES

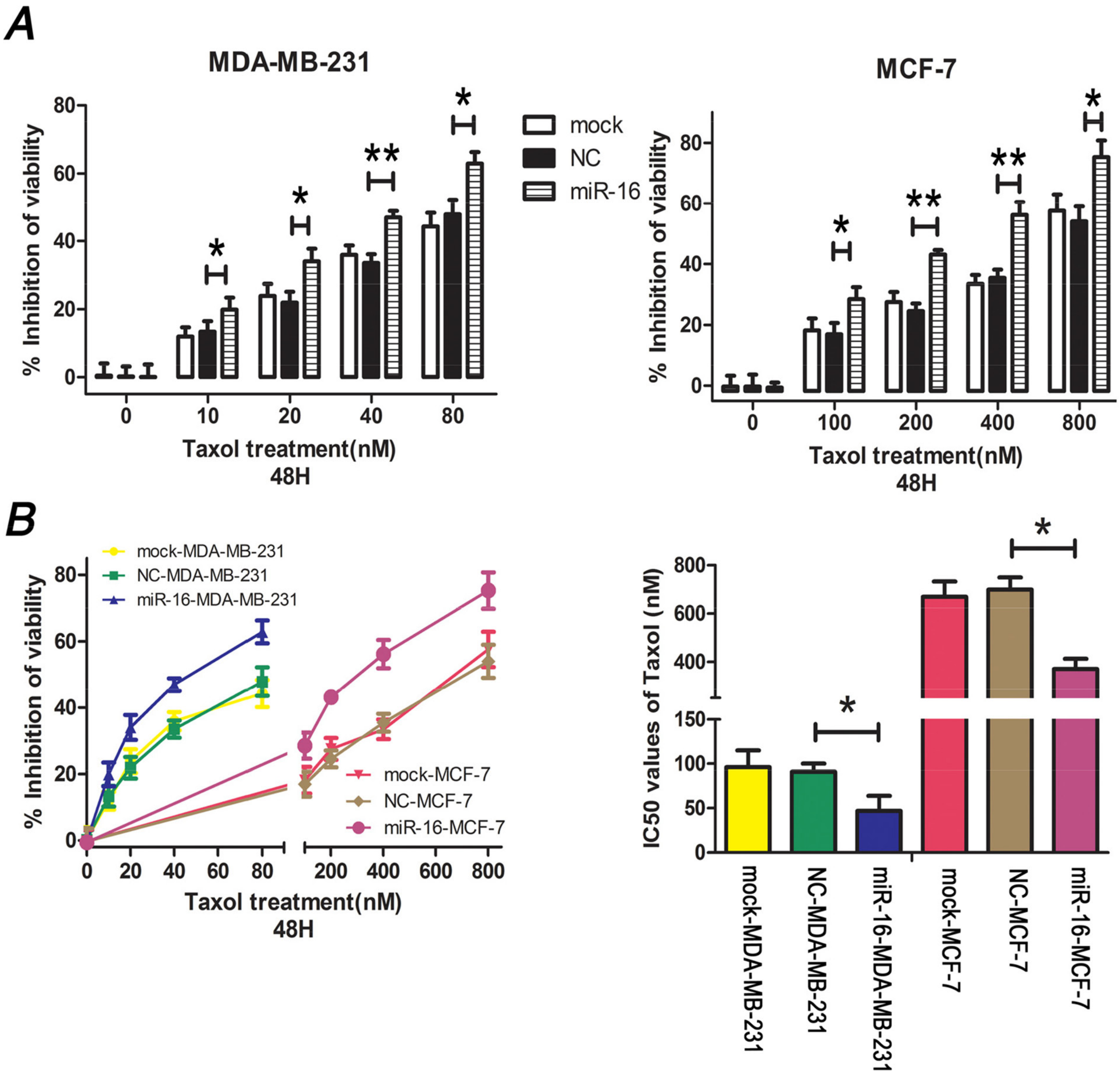

Supplementary Figure S1: Involvement of miR-16 in Taxol chemosensitivity in breast cancer. A-B. MDA-MB-231 and MCF-7 cells mock-transfected or transfected with 50 nM miR-16 mimics or 50 nM miR-NC were seeded into 96-well plates and treated with 0, 10, 20, 40, 80 nM (MDA-MB-231) or 0, 100, 200, 400, 800 nM (MCF-7) Taxol for 48 h. The cell viabilities were detected by MTT assays and the IC50 values were calculated for the three conditions. Data are presented as the percentage of viability inhibition measured in untreated cells. Columns, mean of three independent experiments; bars, SE. *, p<0.05, **, p<0.01.

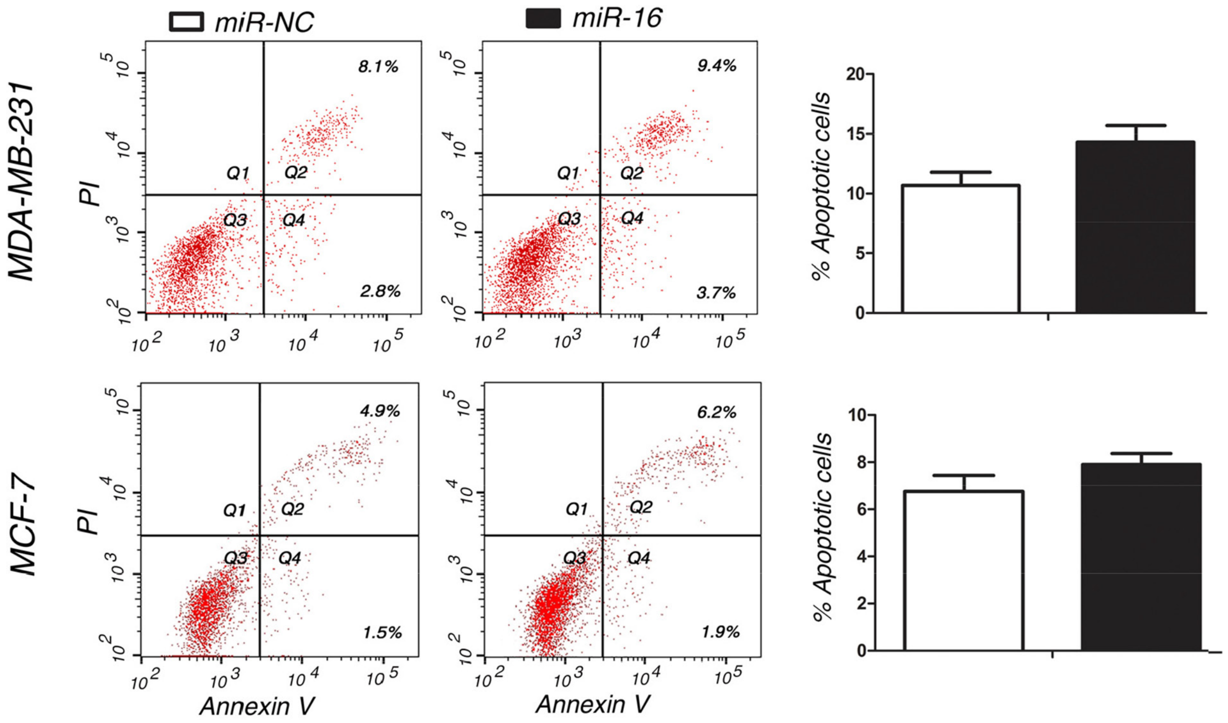

Supplementary Figure S2: Effect of miR-16 on cell apoptosis in breast cancer cell. MDA-MB-231 and MCF-7 cells transfected with 50 nM miR-negative control or miR-16 mimics were collected for annexin V staining and flow cytometry assays. The percentage of apoptotic cells is represented in a bar diagram.

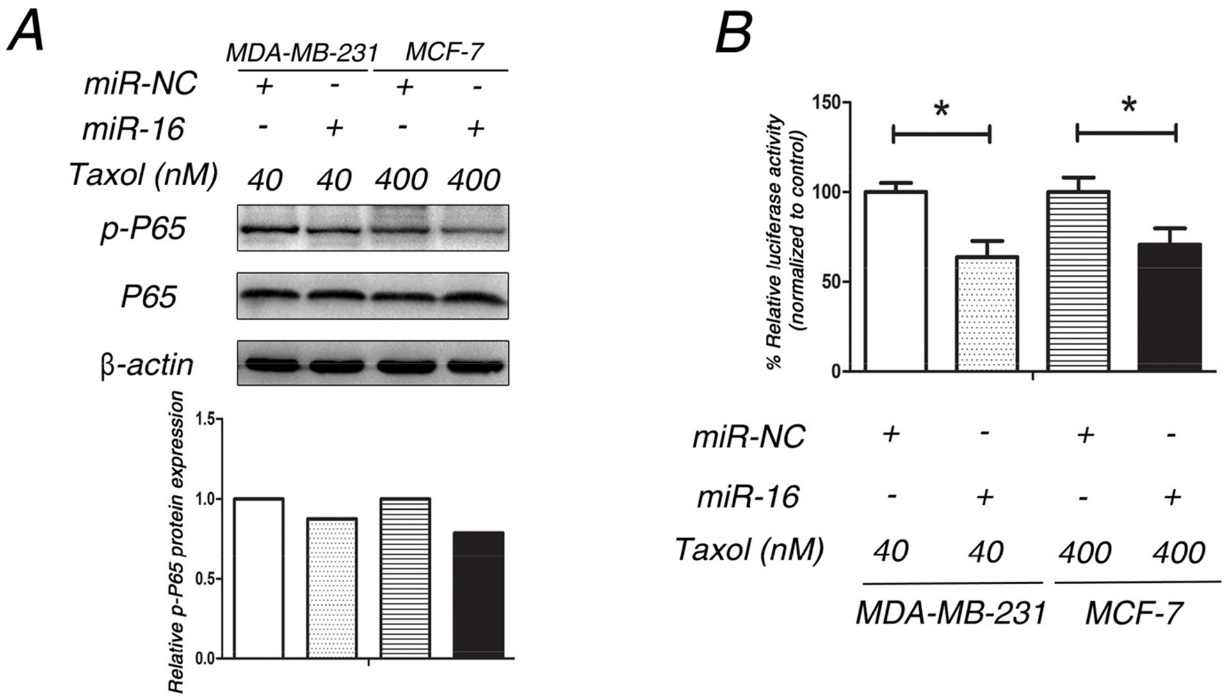

Supplementary Figure S3: NF-κB signalling pathway is inhibited after overexpression of miR-16 with Taxol treatment in breast cancer cells. A. MDA-MB-231 and MCF-7 cells transfected with 50 nM miR-16 mimics or 50 nM miR-NC were treated with 40 nM (MDA-MB-231) or 400 nM (MCF-7) Taxol for 48 h, respectively. Cell lysates were extracted for western blotting using antibodies against P65 and p-P65. β-actin was used as an internal control. The gray density was quantified using the ImageJ software and normalized to P65 and β-actin. **B.** MDA-MB-231 and MCF-7 were co-transfected with 50 nM miR-negative control or miR-16 mimics, pNF-κ B-Luc construct and pRL-TK plasmid (Promega, Madison, WI, USA) using Lipofectamine 3000 reagent. Luciferase activity was measured after treatment with 40 nM (MDA-MB-231) or 400 nM (MCF-7) Taxol for 24 h, respectively. The pRL-TK vector was used as an internal control. The results were expressed as relative luciferase activity (firefly luc/renilla luc). Columns, mean of three independent experiments; bars, S.E. *, $p<0.05$.
